# Some Messages of the War

**Published:** 1916-05-06

**Authors:** 


### The Hospital

The Workers' Newspaper of Administrative Medicine and Institutional Life, Administration, National Insurance and Health.

No. 1560, Vol. LX. SATURDAY, MAY 6, 1916. PRICE ONE PENNY.

### SOME MESSAGES OF THE WAR.

The deeper thoughts about the war are hardly spoken. Certainly the commonplaces of conversation do not reveal them. Of course they exist—sometimes, it may be, in the shape of a vague wonder and questioning, sometimes with a sharply defined personal note of anguish or with a faith which trial does not daunt. But no one speaks out his heart about these things. The silence is partly that of a people largely shy and inarticulate. In part it is the habit of reserve which cherishes personal things as too sacred for expression. Loss and suffering particularly are, to us as a people, best borne in this fashion. They are our own and cannot be shared, or at least as individuals we do not find words for them. It is much the same with great national anxieties and hopes. Sometimes a great poet has sounded the soul of the nation and has put in living words its heart strains and aspirations; and the people feel he has spoken their thought. But, failing this, there is silence, and the mourners go about the streets. So far, neither poet nor prophet has been vouchsafed to us. We appear to have nothing but the politicians and the newspapers. These make noise enough in very truth. But they leave our deeper thoughts unexpressed and our heartfelt questionings unanswered. Instead of quiet convictions they give us wranglings and contentions. A great welcome awaits the genius who can see the true meanings of the trouble of the world and can lighten the gloom with confidence and sure hope. Not that the country has any doubt of ultimate triumph over its enemies. This, however, is in a sense but the superficial and mechanical aspect of our agitations. What is behind the events, and how the tragedy can be reconciled with the smooth sayings of convention—here are the searchings and the difficulties. The returning joys and beauties of Spring force a contrast which few can escape. The leaf and bud are here with all their hopefulness and charm. Yet Death rides triumphant over three continents. Conjure as we will, the hard facts cannot be hidden.

That such thoughts as the above are widely spread is beyond doubt. They are imposed by the facts and are in the very nature of things. In hints and halting utterances they are in part revealed in the literature of the hour. Professional expositors make attempts to satisfy them, but not with any large success. Strictly practical persons affect to disregard them, and urge us to get on with the war. Very good advice too, but getting on with the war for many means suffering and questioning in silence. The mystery and the pain exist whether trenches are lost or won. To triumph over enemies still leaves the questions why we should have enemies and why, to secure the right, the brightness of youth and life must be quenched in blood? Such questions are forced even on the detached observer, and to many, alas! they are sharpened by the sense of personal grief and of loss beyond compensation. Perhaps they cannot be fully answered, but certainly they cannot be suppressed. Doubtless there are some who from temperament or training have a ready solution of all these acute problems, as there are others who are content to leave them in restful and confident mystery. But many have neither one retreat nor the other, and perplexity or even pessimism is their lot.

Yet the situation, even to those who feel all its agitations, is not without its cheerful and encouraging certainties. Even the magnitude of our losses, which mean so much gloom, has not weakened the national resolve, and this, be it remembered, cultivates no selfish or vulgar ambitions. That we are fighting for the defence of what we possess by right is part of our motive no doubt, and the aim is not an unworthy one. But there are also motives which possess a still broader sanction. The claim of the next generation is no small influence. And beyond our own shores are the rights of others, the sense of protection of the weak and the claim of peoples to be free. There is amongst us an energetic conviction that in so far as in us lies justice shall not perish from the earth. Such ideals do not find ready expression in our people, and in the strife of tongues and the noise of affairs they are largely hidden. But it cannot be questioned that they are real and animating influences, and they are operative in all classes. It is no mere lust of victory that has led us to give our sons to wounds and death. It is no common fighting instinct which has urged our artisans to crowd the ranks of battle and to sacrifice rights which they regard as the first protection of their class. There is little phrasing of the motive, but unquestionably moral fervour and a stern sense of justice are foundation influences in the nation's determination. In an age which had been too easily condemned as wholly given over to the idolatry of material comfort men are still ready to suffer and to die for an idea. Amidst all the suffering and bitterness and loss this truth shines prominently, and not even the pessimist may gainsay it. There is still in man a recognition of the nobleness of sacrifice and a scorn of the narrowing blight of selfishness. Those who look at the war with sad eyes must not close them to this brighter vision.

